# Laminar VASO fMRI in focal hand dystonia patients

**DOI:** 10.3389/dyst.2023.10806

**Published:** 2023-02-01

**Authors:** Laurentius Huber, Panagiotis Kassavetis, Omer Faruk Gulban, Mark Hallett, Silvina G. Horovitz

**Affiliations:** 1 Department of Cognitive Neuroscience, Faculty of Psychology and Neuroscience, Maastricht University, Maastricht, Netherlands,; 2 Department of Neurology, University of Utah, Salt Lake City, UT, United States,; 3 Human Motor Control Section, NINDS, NIH, Bethesda, MD, United States,; 4 Brain Innovation, Maastricht, Netherlands

**Keywords:** layer-fMRI, VASO, focal hand dystonia, 7 Tesla, cerebral blood volume fMRI

## Abstract

Focal Hand Dystonia (FHD) is a disabling movement disorder characterized by involuntary movements, cramps and spasms. It is associated with pathological neural microcircuits in the cortical somatosensory system. While invasive preclinical modalities allow researchers to probe specific neural microcircuits of cortical layers and columns, conventional functional magnetic resonance imaging (fMRI) cannot resolve such small neural computational units. In this study, we take advantage of recent developments in ultra-high-field MRI hardware and MR-sequences to capture altered digit representations and laminar processing in FHD patients. We aim to characterize the capability and challenges of layer-specific imaging and analysis tools in resolving laminar and columnar structures in clinical research setups. We scanned N = 4 affected and N = 5 unaffected hemispheres at 7T and found consistent results of altered neural microcircuitry in FHD patients: 1) In affected hemispheres of FHD patients, we found a breakdown of ordered finger representation in the primary somatosensory cortex, as suggested from previous low-resolution fMRI. 2) In affected primary motor cortices of FHD patients, we furthermore found increased fMRI activity in superficial cortico-cortical neural input layers (II/III), compared to relatively weaker activity in the cortico-spinal output layers (Vb/VI). Overall, we show that layer-fMRI acquisition and analysis tools have the potential to address clinically-driven neuroscience research questions about altered computational mechanisms at the spatial scales that were previously only accessible in animal models. We believe that this study paves the way for easier translation of preclinical work into clinical research in focal hand dystonia and beyond.

## Introduction

**FIGURE 1:**
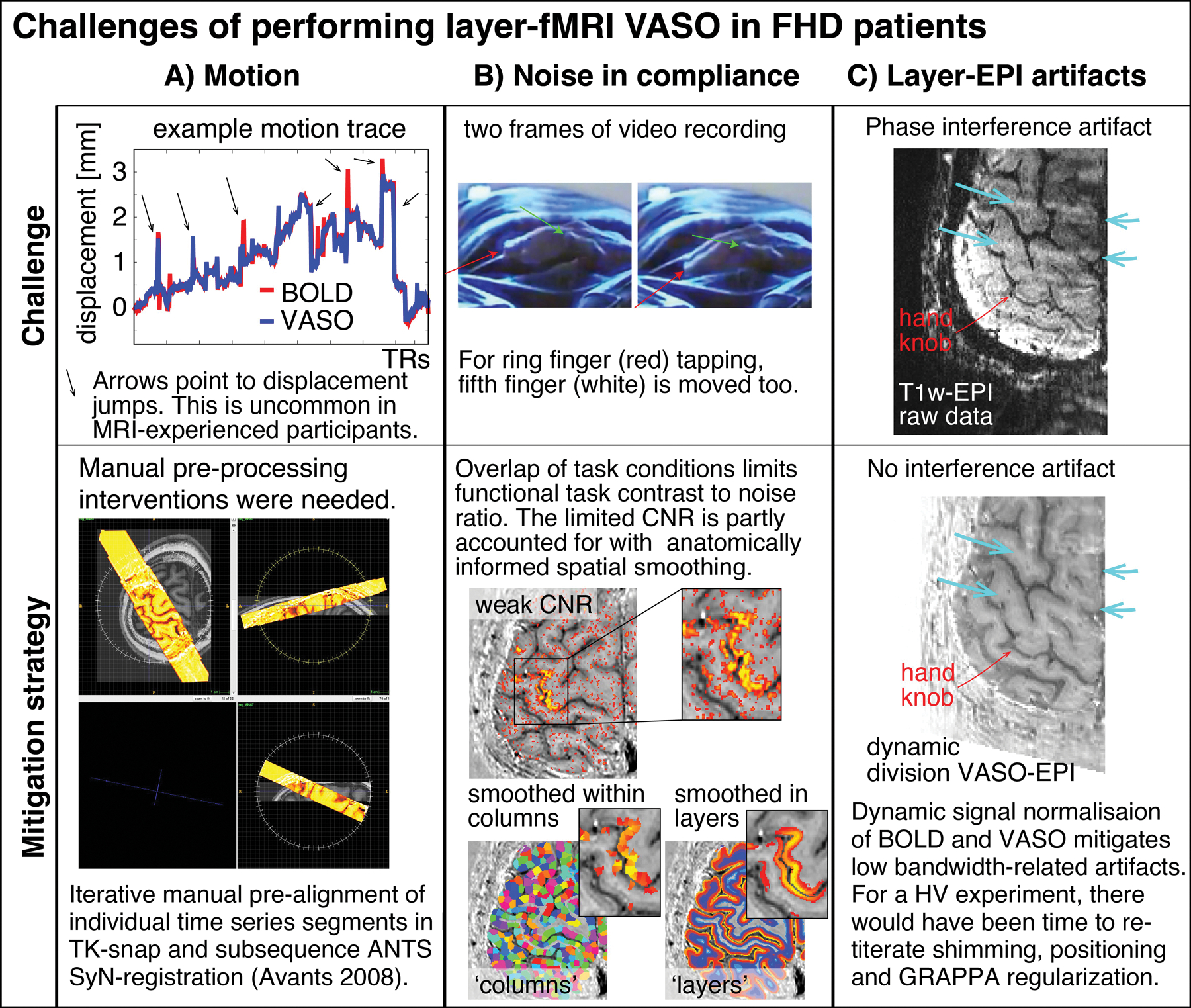
Selected examples of challenging aspects in the acquisition and processing of sub-millimeter VASO fMRI in FHD patients. **(A)** In this study, head motion was mitigated with time consuming manual corrections. **(B)** Limited finger specific functional contrast-to-noise-ratio (CNR) was mitigated with anatomically informed signal pooling within layers and columns, respectively. **(C)** Time constant EPI phase interference artifacts were mitigated by means of dynamic division of odd and even time points with identical artifacts in SS-SI VASO. The purpose of this figure is to exemplify challenges of performing mesoscopic fMRI experiments, which we had not encountered in previous studies. Different from this study, for data collection in previous studies, the sequence-developer had operated the scanner themselves and they had solely scanned experienced healthy volunteers with extraordinary skills to lie perfectly still.

Focal Hand Dystonia (FHD) is a disabling movement disorder characterized by involuntary movements, cramps and spasms. Common sub-forms are known as writer’s cramp and musician’s cramp (Karp 2017) with epidemiological prevalence up to 1 in 2,500 people (1). FHD is associated with pathological neural microcircuits in the cortical somatosensory system. FHD is expected to have closer (1–2 mm) and more overlapping finger representation in the somatosensory system (2–7). While invasive preclinical neuroimaging modalities in animal models allow researchers to probe specific neural microcircuits at the mesoscopic scale of cortical layers and columns (8), conventional non-invasive functional magnetic resonance imaging (fMRI) in humans cannot resolve such small neural computational units. Thus, until now, non-invasive research tools that are routinely applicable in patients, could not yet reach their full potential in translating pre-clinical findings of mesoscopic neural circuitry to patients. Recent developments in ultra-high field MRI hardware and functional contrast generation allows researchers for the first time to capture layer-specific fMRI responses at the spatial scale of sub-millimeter voxels. Specifically, mesoscale fMRI with blood volume sensitive VASO (9, 10) methods can map functional activation changes in the laminar microcircuitry and columnar finger representations without unwanted large draining vein effects of conventional BOLD fMRI (11). For example, previous high-resolution VASO imaging could capture the fine scale finger movement representations across layers and columns in healthy volunteers (HV) (11, 12). These proof-of-principle studies exploited highly optimized experimental environments that might not be fulfilled with patients (Figure 1). Specifically, submillimeter fMRI in clinical populations as opposed to healthy volunteers might be challenged by the following constraints.

- **Scan duration:** Previous studies could collect up to 18 h of fMRI data per participant. This was achieved by inviting the participants for up to 10 two-hour scan sessions (12). Such large numbers of data collection sessions are not practical for FHD patients.
- **Head motion:** Access to patients is limited. Thus, the experimenter does not have the luxury to solely invite those individuals that have the ability to lie perfectly still for long periods of time (approx. 2 h). As exemplified in Figure 1A), this is not always the case for MRI-naive participants, such as FHD patients.
- **Noise in task compliance:** FHD patients can have a hard time following the task instructions. For example, when instructed to move the ring finger only, the middle finger and fifth finger are often moved too. This reduces the finger functional contrast-to-noise ratio.
- **EPI Artifact level:** Sub-millimeter fMRI is limited by low bandwidths and small coverages. In previous applications in healthy volunteers, this had been accounted for with iterative online fine tuning of acquisition parameters during the data acquisition phase (iterative B0-shimming, GRAPPA regularization, alignment, readout bandwidth). Within the limited scan time of patients, such fine tuning is not feasible. Thus, the raw functional images can be challenged by EPI phase-inconsistency artifacts as exemplified in Figure 1C.

Due to these challenges, the applicability of laminar and columnar functional imaging with fMRI in patient populations remains unclear. The aim of this study is to characterize and mitigate these challenges. We used the altered digit representation in FHD patients as a testbed to explore the capability of applying layer-fMRI VASO to investigate mesoscopic neural representations in patients. Specifically, we sought to investigate the quantifiability of pathological alterations of the mesoscopic finger representations in FHD patients across cortical depth as well as across topographical arrangements along the cortical sheet.

## Methods

### Patient procedure

Study participants were recruited under the NIH Institutional Review Board protocol 17-N-0126 (ClinicalTrials.gov identifier: NCT03223623). Four FHD patients (2M/2F; 50 ± 4.6 years, disease duration 3 to 20 years) receiving regular botulinum toxin injections were scanned at least 3 months after their last injection. Additionally, data from a prior study (12) were analyzed to control for task speed (N = 2).

### MRI scanner

The functional imaging sequence was implemented on a ‘classic’ MAGNETOM 7T scanner (Siemens Healthineers, Erlangen, Germany) using the vendor-provided IDEA environment (VB17A-UHF). For RF transmission and reception, a single-channel-transmit and 32-channel receive head coil (Nova Medical, Wilmington, MA, United States) was used. The scanner was equipped with a SC72 body gradient coil (maximum effective gradient strength used here: 49 mT/m; maximum slew rate used: 199 T/m/s).

### MRI sequence

Slice-selective slab-inversion (SS-SI) VASO sequence (Huber 2014) was used in combination with a 3D-EPI readout, as previously implemented (13). VASO is a non-invasive fMRI sequence approach that is sensitive to changes in CBV by means of an inversion-recovery contrast generation (10). In VASO fMRI, an inversion-recovery pulse sequence is used to selectively null out blood water magnetization at the time of the image acquisition, while leaving extra-vascular signals for detection. An increase in CBV during task-evoked neural activation is then associated with an overall MR-signal decrease, which in-turn is believed to be proportional to the volume increase of nulled blood. In the specific SS-SI VASO variant, CBV sensitive images are concomitantly acquired with conventional BOLD signals in an interleaved fashion. This means that every other TR (here 2.2s) we acquired CBV-sensitive and BOLD-sensitive images, respectively (this results in a pair TR of 4.4s). More practical information, including sequence diagrams and its specific implementation for classical MAGNETOM 7T SIEMENS scanners is provided in form of a manual here: https://layerfmri.com/ss-si-vaso-sequence-manual/. The optimal sequence parameters were tested and optimized in previous studies (12). In short: No slab-oversampling, slab-excitation profile with a bandwidth-time-product of 25, T1-related blurring mitigation with variable flip angles, FLASH GRAPPA 3, vendor’s GRAPPA reconstruction algorithms (Siemens software identifier: IcePAT WIP 571), partial Fourier (6/8) reconstruction with POCS 8, in-plane resolution 0.75 mm, slice-thickness: 0.89 mm, TE = 24 ms, TR/TI = 2.2/1.1s. The 3D-EPI readout consisted of 24 shots for 24 *k*_*z*_-partitions. This refers to 24 slices in image space. The two outermost slices were disregarded in the form of 9.1% oversampling in order to mitigate fold-over artifacts in the second phase encoding direction. This results in 22 slices for further processing and a usable slab thickness of 19.2 mm. The overall field of view after oversampling in both phase encoding directions was 122 mm × 162.6 mm × 19.8 mm. A full list of protocol parameters is available on Github: https://github.com/layerfMRI/Sequence_Github/blob/master/FocalHandDystonia/FHD.pdf. The sequence binaries are freely available *via* the SIEMENS sequence ‘Appstore’ in Teamplay (https://teamplay.siemens.com/).

### Scanning

The VASO imaging slab was positioned to be approximately perpendicularly oriented to the main surface of the central sulcus of one hemisphere. This was achieved by tilting it in two directions. Scanning was conducted using preset parameters for layer-fMRI VASO experiments, without individualized settings to optimize the time of the patients in the scanner. This scanning setup can be used to exemplify the usability and scalability of the experimental procedures beyond specialized MRI-development research groups. No specific custom hardware was necessary during the scanning, beyond the common 7T MRI configuration that is available at >100 centers worldwide. We scanned N = 4 patients and N = 2 healthy volunteers. Together, we collected data from N = 5 healthy hemispheres and N = 4 affected hemispheres.

### Task

For the sake of consistency and comparability with previous studies, we used the same tapping tasks as previously used (Huber 2020). Briefly, before each run, subjects were instructed on what hand to tap with. Participants were instructed to tap one finger by extension-flexion at the metacarpophalangeal joint. The same tapping task was repeated for each finger. The tapping frequency was self-paced at a frequency of approximately every 1–2 s. During the scan, a video prompted which finger to move and when to rest. The tapping timing was locked to scanner triggers in units of 16 TRs (2.2 s each) and contains visual cues when to tap which finger and for how long. The task was controlled *via* Psychopy 2 with publicly available scripts (https://github.com/layerfMRI/Phychopy_git/tree/master/Tapping_withTR_all_fingers). The same task was repeated for each hand. Each run lasted approximately 33 min.

### Processing

Concomitantly acquired time series consisting of blood-nulled and BOLD contrasts were separately corrected for motion using SPM12 (Functional Imaging Laboratory, University College London, United Kingdom). Motion-corrected time series were corrected for BOLD contaminations, by means of dynamically dividing blood-nulled signals with not-nulled BOLD signals using LayNii’s v2.2.1 (14) LN_BOCO. In order to mitigate non-steady state effects (transients of hemodynamic response), the division was performed on a two-fold temporally upsampled time series. This form of BOLD correction in SS-SI VASO has been originally been developed, described, and validated for 7T imaging (15) and is based on the assumption that the VASO *T*_1_-contrast (in the *M*_*z*_-direction) is completely orthogonal to the BOLD $T_{2}^{*}$ (in the *M*_*xy*_-direction). Block design activation z-scores and beta estimates were extracted with FSL-FEAT (16). Layerification and columnification were done with the LN2_LAYERS program in LayNii. The motivation and the working principle of the underlying algorithms are explained here https://layerfmri.com/equivol/ and here https://thingsonthings.org/ln2_layers/, respectively.

Hands-on tutorials (with maintained QnA sections), analysis manuals, and “click-along” video instruction of the entire preprocessing and layerification analysis that is performed here can be found at https://layerfmri.com/analysispipeline/.

Cortical patch flattening was performed with the LN2_MULTILATERATE and LN2_PATCH_FLATTEN programs in LayNii (17). The algorithms and how we practically applied them is illustrated here https://thingsonthings.org/ln2_multilaterate/ and here https://youtube.com/playlist?list=PLs_umVHtShfadNm8brOweXHUSmqVDTk4q, respectively. Layer-extraction was manually constrained to the Brodmann area BA4a. This is the evolutionary older part of the primary motor cortex (as opposed to BA4p). In order to pinpoint it, we followed previously described landmarks (11). In short: we located the lateral part of the hand knob as the location on the precentral gyrus with the shortest curvature radius and selected the cortical patch medial to it. Further information of the landmarks with a collection of anatomical examples and instructions of the placement of the fMRI slab and area selection is available here: https://layerfmri.com/finding-roi-of-the-double-layers-in-m1/. The specific ROIs of all patients and hemispheres as used in this study are shown in Figure 4C.

### Re-analysis from previously published studies

We collected short video recordings of the FHD patients while they were performing the tapping task (example screenshots in Figure 1B). This video material suggested a qualitative trend that FHD patients might have performed the tapping at a slower pace than what we were used to from previous studies with the same task. In order to quantify the effect of the tapping frequency on the interpretability of the results of this study, we obtained previously acquired layer-fMRI VASO data that were recorded with varying tapping frequencies. As described in (13), the resolution was 0.75 mm × 0.75 mm × 1.2 mm, with VASO, at 7T in healthy participants. The tapping frequencies were 2 and 0.25 Hz, which covers the range of tapping frequencies that we saw in FHD patients.

## Results

**FIGURE 2:**
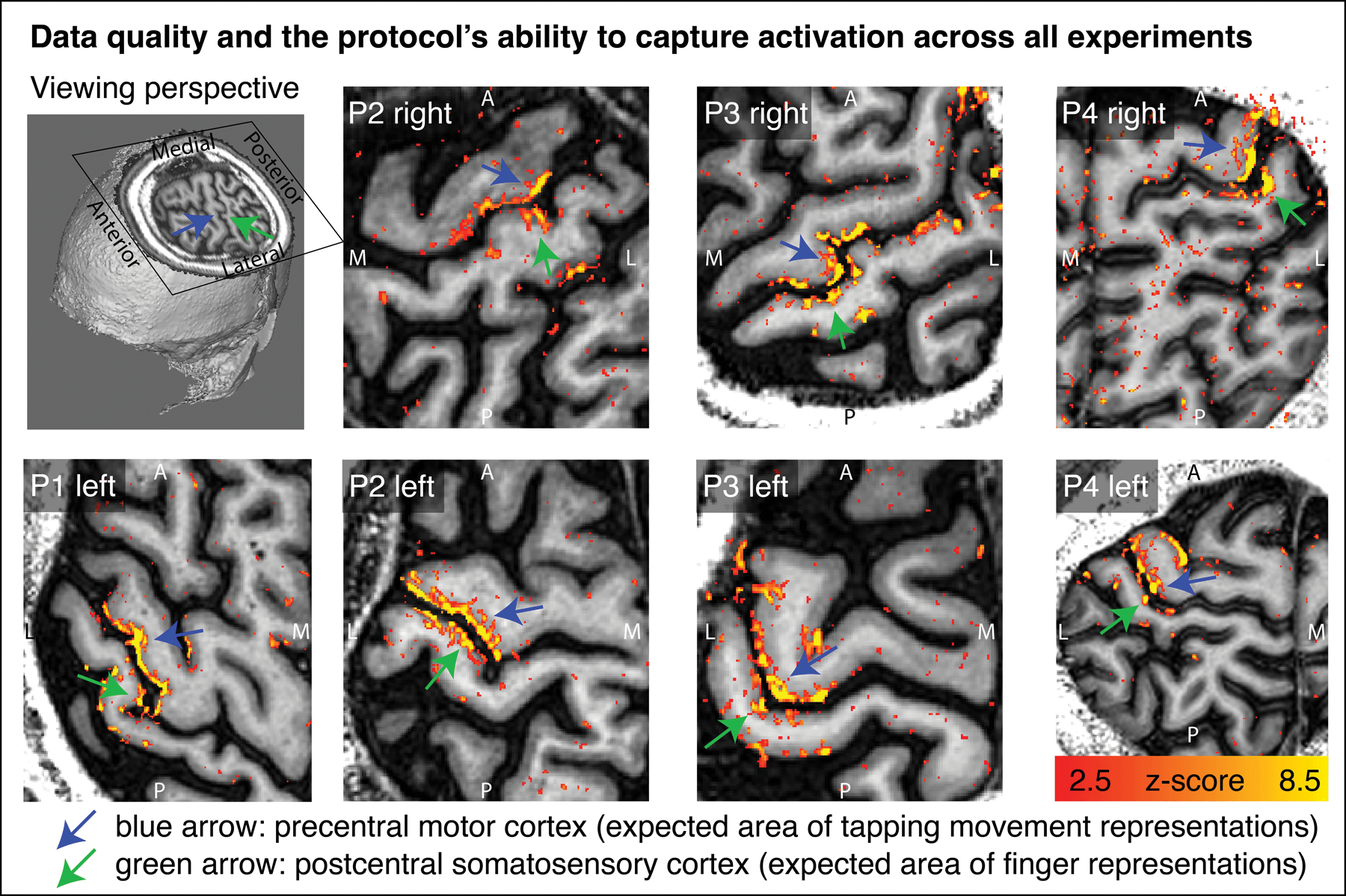
Tapping induced activation maps across patients. Within the 33 min functional experiments, enough data are obtained to extract significant VASO signal changes across all patients and hemispheres. The figures represent the signal without spatial smoothing in spatially upsampled in-plane resolution of 0.4 mm (nominal resolution 0.75 mm). The purpose of this figure is to show that the imaging protocol tested here is capable of capturing blood volume at sub-millimeter resolutions within 33 min functional experiments. We see significant activation in each and every patient and hemisphere.

Across all data sets, we find tapping induced activation both in M1 and in S1. This means that five out of five patient hemispheres show clear CBV changes in M1 and also four out of four patient hemispheres show clear CBV changes in S1. This is a success rate of 100% for both brain areas. Activation maps of all hemispheres in Figure 2 shows that the experimental sub-millimeter VASO imaging setup can reliably capture neurally-induced functional cerebral blood volume (CBV) changes in patients across hemispheres and individuals. It can be seen how the activity is confined to gray matter (GM) without the sensitivity of large drain pial veins above the GM surface.

**FIGURE 3:**
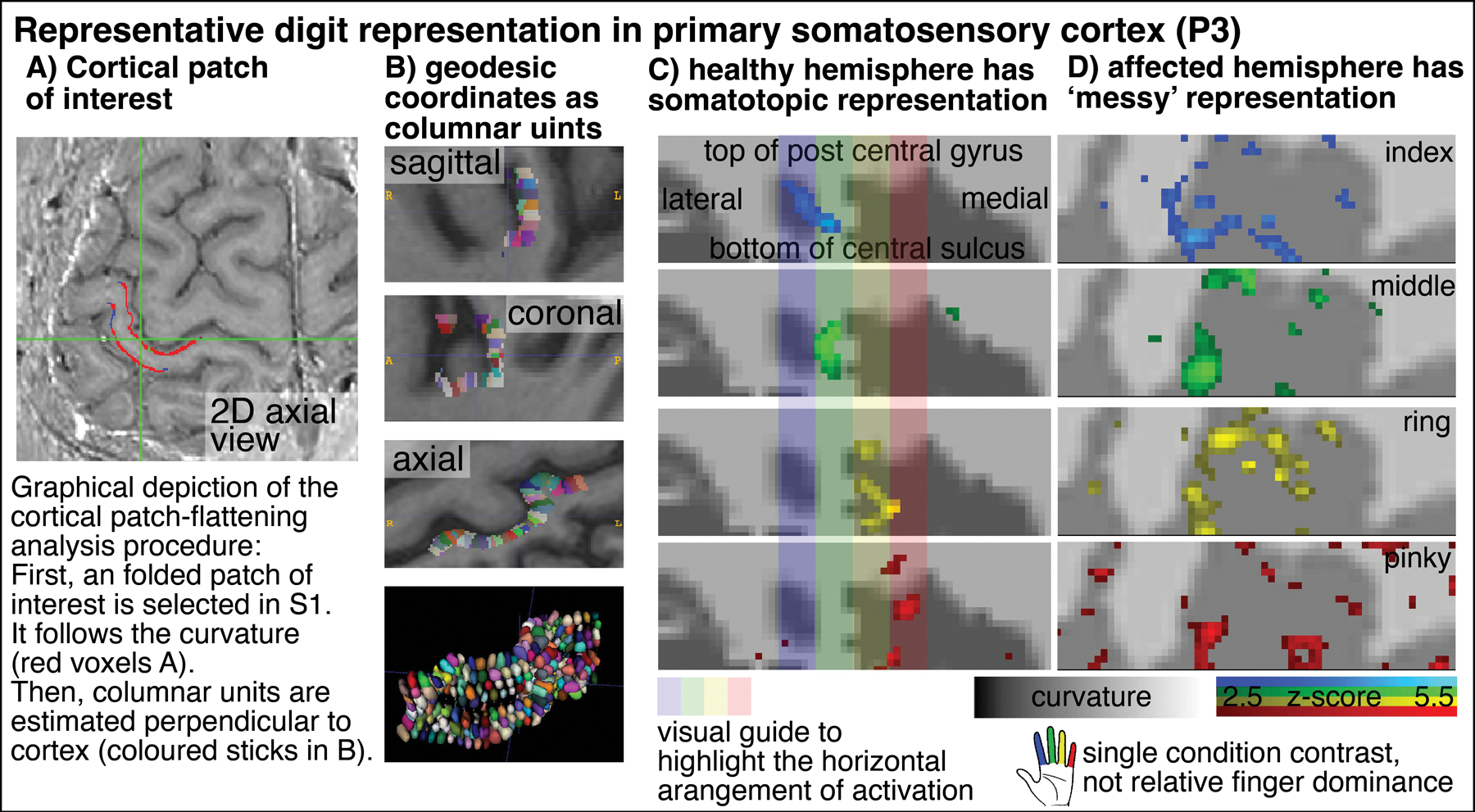
Cortical flattening of thin MRI slab Columns **(A,B)** exemplify the analysis procedure of the cortical flattening. With the methods developed here, the flattening is possible despite the fact that the thin slab does not fulfill common topology requirements that are necessary in mesh-based analyses. Here, we imposed a local coordinate system in the distorted EPI data with LN2_MULTILATERATE (17). More explanations of the underlying algorithms are explained here https://thingsonthings.org/ln2_multilaterate/. Representative VASO-fMRI finger responses in panel **(C,D)** show somatotopic alignment in the healthy hemisphere and less so in the affected hemisphere (smoothed across layers only, no smoothing in the lateral direction). The activation patterns in panel **(C,D)** refer to the average signal across all cortical depths. In this figure, laminar signal distributions are not resolved. The purpose of this figure is to exemplify that sub-millimeter fMRI methods have the potential to address research questions of topographical neural representations in the sub-millimeter spatial scale.

In order to explore the spatial structure of finger representation in the primary somatosensory cortex on the posterior bank of the central sulcus, we imposed a local coordinate system within the GM cortical sheet. Exemplary data of one representative participant show exemplary how the finger representation follows the somatotopic alignment for the healthy hemisphere (Figure 3). The spatial structure of the representations in the FHD-affected hemisphere are less clear. This is not for the lack of detection sensitivity. In fact, large patches of the primary somatosensory cortex are clearly engaged during finger tapping tasks. It’s just that the spatial arrangement of the finger-specific activation patches does not follow as clear arrangements as for the healthy hemisphere.

**FIGURE 4:**
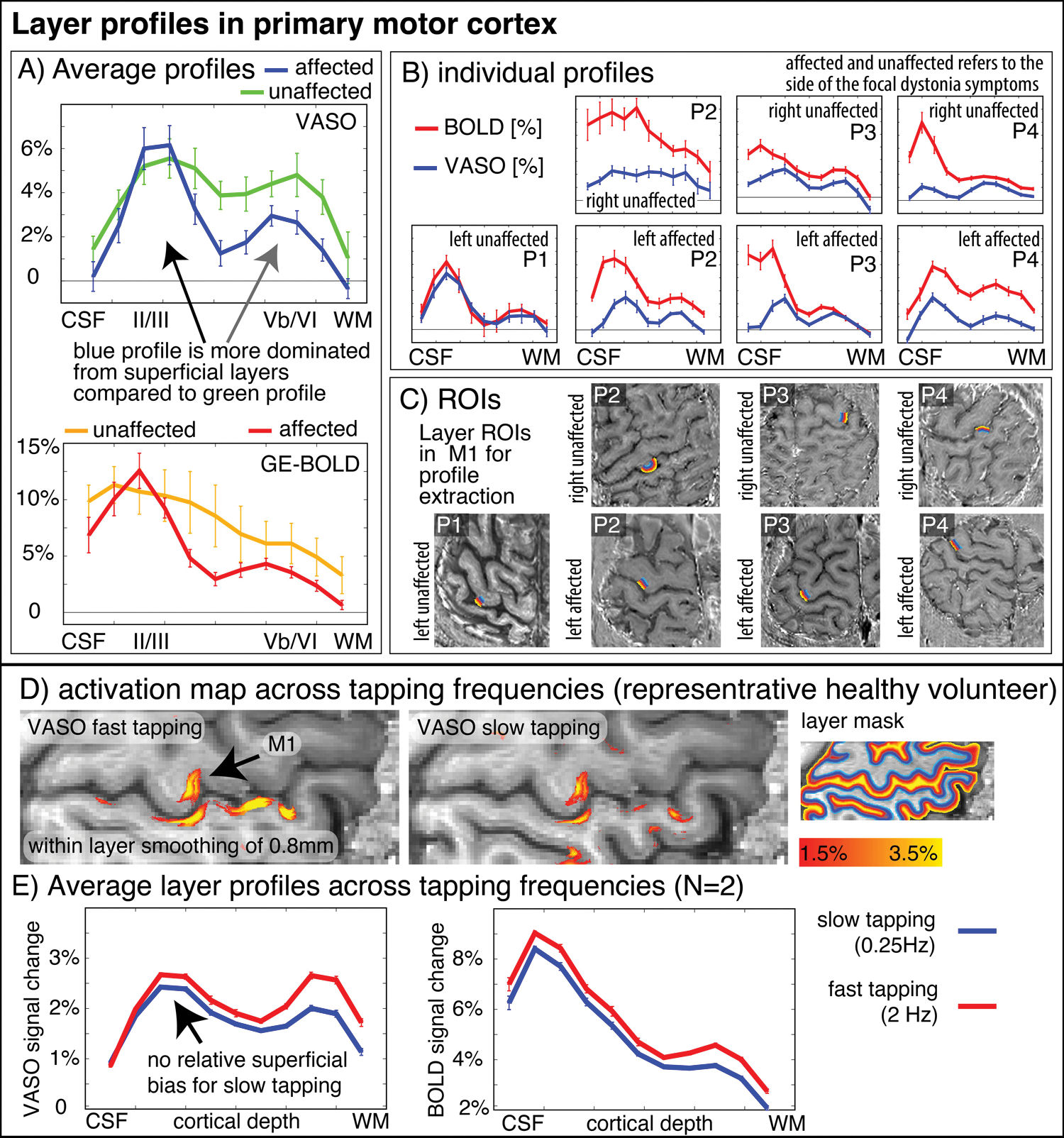
Layer-fMRI profiles in the primary motor cortex for BOLD and VASO in healthy and affected hemispheres. Panels **(A,B)** highlight how all layer profiles consistently show a larger superficial bias in GE-BOLD compared to VASO, while still showing clear indication of a secondary “bump” in the deeper output layers [gray in **(A)**]. Compared to the deeper layers, the superficial layers in the affected hemispheres seem to be relatively stronger activated compared to their healthy counterparts (see black arrow compared to gray arrow). Signal pooling of layers was done from unsmoothed data. The corresponding ROIs are depicted in Panel **(C)**. Note that the layer profiles depicted here are referring to data that are collapsed across the columnar direction. This means that the analysis procedure shown here does not represent topographical signal distributions along the cortical ribbon. Panels **(D,E)** show layer-dependent CBV responses across tapping frequencies in healthy participants. It can be seen that slower tapping results in overall smaller fMRI signal changes. The relative reduction of fMRI signal changes is stronger in the deeper “output” layers compared to the superficial “input” layers. This suggests that the layer-specific signal modulation between healthy and affected hemispheres in Panel **(A)** cannot be explained by different tapping frequencies. The purpose of this figure is to exemplify the potential of sub-millimeter methodology to address questions of alterations in laminar processing. Furthermore, it is shown how consistent the results are across a small sample of patients.

Aside from topographical finger-representations in the sensory cortex, we also explored the laminar blood volume responses in the primary motor cortex. Figure 4 depicts M1 layer-profiles of all patients. As expected, GE-BOLD signals show an increased bias towards the superficial layers compared to CBV-sensitive VASO. This is manifested in steeper slopes of the BOLD profiles compared to the VASO profiles in all hemispheres, in all patients, and in all functional tasks (100% of a total of N = 14), without exceptions. The affected hemispheres seem to be more dominated by superficial cortico-cortical input-layers compared to the healthy hemispheres, in all participants (100% of N = 4).

Video material of the FHD patients performing the task inside the scanner bore suggested a qualitative trend that FHD patients might have performed the tapping at a slower pace compared to typical tapping frequencies in healthy participants. To quantify the effect of tapping frequency on the interpretation of the layer profiles shown in Figure 4A, we also show the tapping frequency dependence of the layer profile as collected in previous studies. These results are shown in Figure 4E and suggest that a potentially different tapping frequency cannot explain the layer-dependent fMRI signal modulations that we find in healthy vs. affected hemispheres.

## Discussion

In this study, we explored the capabilities and challenges of high-resolution layer-fMRI to inform research questions of affected cortical microcircuitry in patients. We scanned N = 4 focal hand dystonia patients and found consistently altered somatotopic and laminar activation patterns in affected and unaffected hemispheres. The results presented here suggest that the high-resolution acquisition and analysis protocols developed here allow clinical neuroscientists to investigate pathological laminar pathologies in patients. Specifically, we found that cortical hemispheres that were affected by FHD appear to have a less structured somatotopic alignment of finger representation in the primary somatosensory homunculus of the postcentral gyrus. These results are consistent with the parallel ongoing imaging efforts of high-resolution functional mapping of finger representations at 7T (18). This study had already overcome some challenges and mapped the finger representation in FHD patients with 1.5 mm GE-BOLD (18). At lower spatial resolutions of 2.4 mm, no alterations of finger representations in the primary somatosensory cortex could be detected (19). Here, we pushed the imaging protocols further and used sub-millimeter vein-bias-free VASO to capture laminar fMRI modulations. Aside from the altered somatotopic topographical digit representations in the primary sensory cortex, we also explored layer-dependent fMRI response in the primary motor cortex. Across all participants, we found that the superficial cortico-cortical input layers seem to have an increased activation during finger tapping tasks compared to unaffected hemispheres while the opposite is true for deeper layers. The increased activation may be due to decreased inhibition, and, in this way, these findings would be consistent with the notion that FHD is associated with a lack of neural surround-inhibition mechanisms (6, 20–22). However, the relative dominance of fMRI signal changes in input layers could also be partly related to increased mental load (attention, motor planning, incorporating sensory feedback) and corresponding cortico-cortical input that is expected in the superficial layers (23–25). While the tapping frequency might have been slower for affected and unaffected hemispheres, we do not think that this can influence the interpretability of the layer-dependent activity modulation. In fact, slower tapping frequencies are expected to result in weaker superficial activation (Figure 4E), which we do not see in the results of FHD affected hemispheres.

### Relevance of this work

Looking beyond FHD, the tools developed and tested in this study provide a starting point for mapping layer-specific connections in patient populations in the general context of clinical neuroscience. Many influential theories of brain function of disorders posit hypotheses of neural deficits in distinct cortical layers and their role of feedforward and feedback processing. For example: mental disorders, such as autism and schizophrenia, and neurodegenerative diseases such as Parkinson’s or Huntington’s disease. Specifically.

- Layer-fMRI VASO has been discussed to be the key future technology to probe predictions of axonal loss and microcircuit dysfunction to provide insights for neurodegenerative motor disease (26, 27). The results shown there suggest that the imaging and analysis methodology is now ready to be applied for such proposed studies.
- Layer-fMRI has been discussed to be a key future technology to act as Occam’s razor for multiple competing hypotheses of hierarchical microcircuit disruptions in psychosis (28, 29). Namely in the context of predictive coding, one theory about psychosis discusses delusion symptoms in terms of deficits in learning. This is associated with respective predicted pathological neural computations in feedback dominated superficial and deeper layers. Another theory of psychosis discusses the same hallucination symptoms in terms of deficits in perceptual inference with respective predicted pathological neural computations in feedforward dominated middle layers. Until now, the methodological ability to constrain the models with empirical data has been limited. The usability of the layer-fMRI VASO protocols developed here allows future clinical neuroscientists to test and thus constrain these models.

These examples show that layer-fMRI applications in patients open the door to investigating computational mechanisms at the spatial scales that were previously only accessible in animal models. We believe that this study paves the way for easier translation of preclinical work into clinical research in focal hand dystonia and beyond.

### In conclusion

While there are plentiful review articles about the value of layer-fMRI for clinical neuroscience research (26–29) experimental layer-fMRI data are scarce (30). Here, we present fMRI results of laminar and columnar CBV changes in FHD patients. We could not confirm previous findings of closer finger representations (2, 4, 5) because the finger representations in our maps did not show clear locations from which the distances could be calculated. We find that FHD affected hemispheres had a relatively stronger activity in motor input-layers (II/III) compared to the activity in motor output-layers (Vb/VI). This might be due to reduced surround inhibition in the superficial layers of the affected hemispheres. It could also be related to increased mental load (attention, motor planning, incorporating sensory feedback) that is related to neural input terminating in superficial layers. More data are needed to ultimately determine the stability of this finding. This study has built the methodological groundwork that future clinically-focused neuroscience application studies of layer-fMRI can be based on.

## Data Availability

The datasets generated for this study are deposited in the NIH.BOX / data.ninds.gov repository https://layerfmri.page.link/FHD_data. Access is granted individually without conditions and should be requested from the corresponding author silvina. horovitz@nih.gov.

## References

[1] Torres-Russotto D, Perlmutter JS. Focal dystonias of the hand and upper extremity. J Hand Surg Am (2008) 33(9):1657–8. doi:10.1016/j.jhsa.2008.09.001 18984354 PMC2662617

[2] Bara-Jimenez W, Catalan MJ, Hallett M, Gerloff C. Abnormal somatosensory homunculus in dystonia of the hand. Ann Neurol (1998) 44(5):828–31. doi:10.1002/ana.410440520 9818942

[3] Butterworth S, Francis S, Kelly E, McGlone F, Bowtell R, Sawle GV. Abnormal cortical sensory activation in dystonia: An fMRI study. Mov Disord (2003) 18(6): 673–82. doi:10.1002/mds.10416 12784271

[4] Catalan MJ, Ishii K, Bara-Jimenez W, Hallett M. Reorganization of the human somatosensory cortex in hand dystonia. J Movement Disord (2012) 5(1):5–8. doi:10.14802/jmd.12002 PMC4027675 24868405

[5] Elbert T, Candia CAV, Altenmüller E, Rau H, Sterr A, Rockstroh B, Alteration of digital representations in somatosensory cortex in focal hand dystonia. NeuroReport (1998) 9(16):3571–5. doi:10.1097/00001756-199811160-00006 9858362

[6] Hallett M Neurophysiology of dystonia: The role of inhibition. Neurobiol Dis (2011) 42(2):177–84. doi:10.1016/j.nbd.2010.08.025 20817092 PMC3016461

[7] Nelson AJ, Blake DT, Chen R. Digit-specific aberrations in the primary somatosensory cortex in writer’s cramp. Ann Neurol (2009) 66(2):146–54. doi:10.1002/ana.21626 19743446

[8] Goense J, Bohraus Y, Logothetis NK. fMRI at high spatial resolution: Implications for BOLD-models. Front Comput Neurosci (2016) 10():66–14. doi:10.3389/fncom.2016.00066 27445782 PMC4923185

[9] Hua J, Jones CK, Qin Q, Van Zijl PC. Implementation of vascular-space-occupancy MRI at 7T. Magn Reson Med (2013) 69(4):1003–13. doi:10.1002/mrm.24334 22585570 PMC4121129

[10] Lu H, Golay X, Pekar JJ, Van Zijl PCM. Functional magnetic resonance imaging based on changes in vascular space occupancy. Magn Reson Med (2003) 50(2):263–74. doi:10.1002/mrm.10519 12876702

[11] Huber L, Handwerker DA, Jangraw DC, Chen G, Hall A, Stüber C, High-resolution CBV-fMRI allows mapping of laminar activity and connectivity of cortical input and output in human M1. Neuron (2017) 96(6):1253–63. doi:10.1016/j.neuron.2017.11.005 29224727 PMC5739950

[12] Huber L, Finn ES, Handwerker DA, Bönstrup M, Glen DR, Kashyap S, Sub-millimeter fMRI reveals multiple topographical digit representations that form action maps in human motor cortex. NeuroImage (2020) 208:116463. doi:10.1016/j.neuroimage.2019.116463 31862526 PMC11829252

[13] Poser BA, Koopmans PJ, Witzel T, Wald LL, Barth M. Three dimensional echo-planar imaging at 7 tesla. NeuroImage (2010) 51(1):261–6. doi:10.1016/j.neuroimage.2010.01.108 20139009 PMC2853246

[14] Huber LRR, Poser BA, Bandettini PA, Arora K, Wagstyl K, Cho S, LayNii: A software suite for layer-fMRI. NeuroImage (2021) 237:118091. doi:10.016/j.neuroimage.2021.118091 33991698 PMC7615890

[15] Huber L, Ivanov D, Krieger SN, Streicher MN, Mildner T, Poser BA, Slab-selective, BOLD-corrected VASO at 7 tesla provides measures of cerebral blood volume reactivity with high signal-to-noise ratio. Magn Reson Med (2014) 72(1):137–48. doi:10.1002/mrm.24916 23963641

[16] Jenkinson M, Beckmann CF, Behrens TEJ, Woolrich MW, Smith SM. Fsl. NeuroImage (2012) 62:782–90. doi:10.1016/j.neuroimage.2011.09.015 21979382

[17] Gulban OF, Bollmann S, Huber R, Wagstyl K, Goebel R, Poser BA, Mesoscopic quantification of cortical architecture in the living human brain. Neuroimage (2022). 10.1016/j.neuroimage.2022.119733 36375782

[18] Pakenham D, Asghar M, Glover P, O’Neill G, Sengupta A, Schluppeck D, Assessing somatotopic and mototopic organisation in Focal Hand Dystonia using high-resolution 7T fMRI. In: Proc. Joint Annual Meeting ISMRM (2019). p. 0361.

[19] Sadnicka A, Wiestler T, Butler K, Altenmüller E, Edwards M, Ejaz N, Intact finger representation within primary sensorimotor cortex of musician’s dystonia. Brain (2022) awac356. page awac356. doi:10.1093/brain/awac356 36170332 PMC10115231

[20] Brüggemann N Contemporary functional neuroanatomy and pathophysiology of dystonia. J Neural Transm (2021) 128:499–508. doi:10.1007/s00702-021-02299-y 33486625 PMC8099808

[21] Gallea C, Herath P, Voon V, Lerner A, Ostuni J, Saad Z, Loss of inhibition in sensorimotor networks in focal hand dystonia. NeuroImage: Clin (2018) 17:90–7. doi:10.1016/j.nicl.2017.10.011 29062685 PMC5645005

[22] Kassavetis P, Sadnicka A, Saifee TA, Pareés I, Kojovic M, Bhatia KP, Reappraising the role of motor surround inhibition in dystonia. J Neurol Sci (2018) 390:178–83. doi:10.1016/j.jns.2018.04.015 29801882

[23] Persichetti AS, Avery JA, Huber L, Merriam EP, Martin A. Layer-specific contributions to imagined and executed hand movements in human primary motor cortex. Curr Biol (2020) 30(9):1721–5. doi:10.1016/j.cub.2020.02.046 32220318 PMC7202997

[24] Trampel R, Bazin P-L, Schäfer A, Heidemann RM, Ivanov D, Lohmann G, Laminar-specific fingerprints of different sensorimotor areas obtained during imagined and actual finger tapping. Proc Intl Soc Mag Reson Med (2012) 20:663.

[25] Turner R Uses, misuses, new uses and fundamental limitations of magnetic resonance imaging in cognitive science. Philosophical Trans R Soc B: Biol Sci (2016) 371:20150349. doi:10.1098/rstb.2015.0349 PMC5003851 27574303

[26] McColgan P, Joubert J, Tabrizi SJ, Rees G. The human motor cortex microcircuit: Insights for neurodegenerative disease. Nat Rev Neurosci (2020) 21(8):401–15. doi:10.1038/s41583-020-0315-1 32555340

[27] Schreiber S, Northall A, Weber M, Vielhaber S, Kuehn E, McColgan P, Reply to ‘Topographical layer imaging as a tool to track neurodegenerative disease spread in M1’. Nat Rev Neurosci (2021) 22(1):69. doi:10.1038/s41583-020-00405-9 33154582

[28] Haarsma J, Kok P, Browning M. The promise of layer-specific neuroimaging for testing predictive coding theories of psychosis. Schizophrenia Res (2020) 245: 68–76. doi:10.1016/j.schres.2020.10.009 PMC9241988 33199171

[29] Stephan KE, Petzschner F, Kasper L, Bayer J, Wellstein K, Stefanics G, Laminar fMRI and computational theories of brain function. NeuroImage (2019) 197:699–706. doi:10.1016/j.neuroimage.2017.11.001 29104148

[30] Wan Y, Qian C, Wen W, Zhang P. (2021). Layer-dependent amblyopic deficits in feedforward and lateral processing in human early visual cortex. In OHBM, page 2632.

